# Association between hemoglobin A1c, Vitamin C, and microbiome in diabetic foot ulcers and intact skin: A cross‐sectional study

**DOI:** 10.1002/hsr2.718

**Published:** 2022-08-03

**Authors:** Khanh Phuong S. Tong, Stefan J. Green, Jacquelyn Ortiz, Stephanie C. Wu

**Affiliations:** ^1^ Center for Lower Extremity Ambulatory Research (CLEAR), Dr. William M. Scholl College of Podiatric Medicine at Rosalind Franklin University of Medicine and Science North Chicago Illinois USA; ^2^ Department of Internal Medicine, Division of Infectious Diseases Rush University Medical Center Chicago Illinois USA

**Keywords:** diabetic foot, glycemic control, microbiome, ulcer, Vitamin C

## Abstract

**Background and Aims:**

Diabetic foot ulcers (DFUs) add billions of dollars to the direct annual costs associated with diabetes. Despite various treatments, many DFUs do not heal and become infected. Both skin‐associated microbial communities and glycemic control are believed to be important in nonhealing DFUs. Recent studies have linked serum Vitamin C levels with glycemic control and DFUs. This cross‐sectional study assessed skin microbiome in DFUs, intact diabetic skin, and nondiabetic skin to identify correlations between hemoglobin A1c (HbA1c), Vitamin C, and microbial community structure. Correlations between Vitamin C, HbA1c, wound size, and ulcer duration were also determined.

**Methods:**

Participants had their DFUs or intact skin culture swabbed. HbA1c was obtained via point‐of‐care fingerstick testing and serum Vitamin C was obtained via venipuncture. All participants completed a dietary questionnaire. Participants with ulcers were stratified into the controlled (≤8.0%) or uncontrolled (>8.0%) HbA1c group. Analysis of microbial communities was performed via 16S ribosomal RNA (rRNA) gene amplicon sequencing and bacterial load was measured by the domain‐level quantitative polymerase chain reaction of the 16S rRNA gene.

**Results:**

Forty‐two patients were recruited over 6 months. Bacteria from the genera *Staphylococcus* and *Stenotrophomonas* were present in all samples and often dominant, but a shift towards anaerobic pathogenic taxa was observed in ulcers. No global significant differences were observed for HbA1c and Vitamin C levels in the microbial community structure (*R* < 0.013/*p* > 0.375). Bacterial loads were 4–5 orders of magnitude higher in ulcers than in intact skin samples. Bacterial load was not significantly higher in the uncontrolled HbA1c group (*p* = 0.67). Larger wound sizes (*p* = 0.46) were observed in the uncontrolled HbA1c group compared to the control. Lower Vitamin C levels (*p* = 0.002) were observed in the uncontrolled HbA1c group compared to nondiabetic controls.

**Conclusion:**

Understanding the link between Vitamin C and HbA1c and DFU microbiome may aid in new therapies.

## 1 INTRODUCTION

Diabetic foot ulcers (DFUs) add 9 to 13 billion dollars to direct annual costs associated with diabetes.^1^, ^2^ Despite treatment, a high proportion of DFUs become infected, resulting in some form of lower extremity amputation (LEA).^3^ In addition to increased microbial load and pathogenic shift of DFU‐associated microbiomes, clinical factors have been associated with slow healing DFUs, including poor glycemic control^4^ and Vitamin C deficiency.^5^, ^6^ According to Lane et al.,^7^ hemoglobin A1c (HbA1c) levels > 8% are associated with increased likelihood of LEA among patients with DFUs. Hyperglycemia‐induced oxidative stress promotes harmful biofilm formation and can lower overall microbial diversity in DFUs.^8^, ^9^ Conversely, antioxidants like Vitamin C have been found to prevent biofilm formation in vitro,^10^ suggesting that Vitamin C may influence the microbiome in DFUs. In addition, recent studies have noted a protective role of Vitamin C in glycemic control,^11^, ^12^ and thus, Vitamin C supplementation is being utilized as a part of diabetes management.^13^

Recent studies have demonstrated that microbial community structure and clinical factors can independently impair the healing of DFUs, but few studies have examined associations between these factors. Pang et al.^14^ measured significantly different microbial α‐diversity on intact skin in patient groups with a differing duration of diabetes using high‐throughput 16S ribosomal RNA (rRNA) sequencing. Their findings showed dynamic changes in intact skin microbiome during the progression of diabetes, suggesting that the duration of diabetes influences skin microbiota. However, they did not evaluate other, more objective clinical factors that may influence skin microbiota. Gardner et al.^15^ evaluated the association between microbial community structure, HbA1c levels in DFUs, and several other clinical factors, but did not compare microbial community structure in DFUs with that of intact diabetic skin (IDS) and intact nondiabetic skin (NDS). They found that poor glycemic control was associated with ulcers containing a high relative abundance of *Staphylococcus* and *Streptococcus*.

In this cross‐sectional study, we evaluated associations between HbA1c, Vitamin C, and microbial community structure in DFUs, IDS, and NDS. Cultivation‐independent molecular techniques were used to interrogate microbial communities, including bacterial load measurements using quantitative PCR (qPCR), and microbial community structure analysis using 16S rRNA gene amplicon sequencing.

## 2 RESEARCH DESIGN AND METHODS

This cross‐sectional study was approved by the institutional review board (IRB protocol number SCPM19‐167) at Rosalind Franklin University of Medicine and Science (RFUMS), North Chicago, IL. Recruitment was performed between July 2019 and December 2019 at two Rosalind Franklin University Health Clinic (RFUHC) locations in North Chicago and Libertyville, IL. All participants received oral and written information and signed the IRB‐approved informed consent before participating. For the control and DFU groups, patients were eligible for the study if they were male or female, and aged 18 years or older. Patients in the DFU group had a Wagner Grade 1 or 2 DFU. Exclusion factors included active wound infection, untreated osteomyelitis, gangrene, immune‐compromising disease, presence of multiple DFUs, dementia, or impaired cognitive function, and current or history of cancer(s).

Subjects were screened to ensure that they met the inclusion and exclusion criteria. Medical history and a standardized physical examination, including tobacco use, height, and weight, were conducted as part of a regular care visit. For patients with DFU, information was recorded regarding the type and duration of diabetes, and the duration of ulcer. Wound dimensions were measured with a disposable measuring ruler for length, width, and depth. Wound size was calculated using the formula for an ellipse: (length/2) × (width/2) × 3.14. Microbiome analysis was performed by swabbing the DFU post debridement and IDS on the contralateral foot at the equivalent location using a cotton‐tip swab (Ref. # 4473979, Regular Size BP 20 mm Single Wrapped MFG: Copan Flock Technologies Srl) via the Levine technique.^16^ In patients without diabetes or ulcers, intact skin of bilateral feet was swabbed at the equivalent location of the gender‐ and age‐matched patient with DFU. Samples were immediately frozen in collection tubes at −2°C to −5°C and sent to the Genome Research Core (GRC) at the University of Illinois at Chicago (UIC), Chicago, IL for processing. HbA1c was obtained via a point‐of‐care fingerstick (A1CNow+; PTS Diagnostics) by K. P. S. T. or J. O. If HbA1c had already been obtained within the past 3 months, finger‐sticking was deferred, and the value in the medical record was used. Approximately 4 cc of serum was collected via venipuncture from the antecubital fossa using previously described techniques^17^, ^18^ for Vitamin C testing and centrifuged in‐house by K. P. S. T. according to the Quest Diagnostics protocol. The serum component was transferred into a separate clean, plastic, and light‐protected transport tube and frozen before pick‐up within 48 h by Quest Diagnostics.

In addition, self‐reported fruit and vegetable (FV) intake was assessed using a 12‐question dietary assessment survey (Appendix A). Patients were asked to report the frequency of consumption of fruits and vegetables on a nine‐point scale ranging from “never” to “2 or more times per day.”

### 2.1 Patient stratification

Patients with diabetes were stratified into two groups: controlled HbA1c (cHbA1c) if values were ≤8.0% or uncontrolled HbA1c (uHbA1c) if values were >8.0%. Normal or in‐range (IR) Vitamin C values were 0.3–2.7 mg/dl for females and 0.2–2.1 mg/dl for males according to the Quest Diagnostics reference ranges. Patients were considered out‐of‐range (OOR) if Vitamin C values were below the lower limit of 0.3 mg/dl for females and 0.2 mg/dl for males.

### 2.2 Microbial community characterization

Genomic DNA was extracted from the skin and DFU culture swabs using a Maxwell16 device (Promega) using a Buccal Swab Low Elution Volume DNA purification kit. Modifications to the default workflow included a lysozyme incubation (10 ng/µl lysozyme; Thermo Fisher Scientific) for 30 min at 37°C, followed by bead‐beating (40 s at 6 m/s) using a FastPrep‐24 System (MP Biomedicals). Homogenized samples were transferred to the Maxwell cartridges for the final purification of DNA. Microbial 16S rRNA gene abundance was quantified using quantitative real‐time PCR, as described previously.^19^ Primers, probes, and double‐stranded synthetic DNA standards (gBLOCKs) were synthesized by Integrated DNA Technologies. Analysis was performed using a ViiA7 Real‐Time PCR instrument (Thermo Fisher Scientific) and with an 8‐order of magnitude standard dilution series for absolute quantification. Genomic DNA was also used as a template for amplification of microbial 16S rRNA gene amplicons using a two‐stage PCR protocol as described previously.^20^ The primer set 515F modified and 806R modified,^21^ targeting the V4 variable region of microbial 16S rRNA genes, was employed,^21^, ^22^, ^23^ and libraries were sequenced on an Illumina MiniSeq instrument, employing paired‐end 2 × 153 base reads. DNA extraction, library preparation, and sequencing were performed in the GRC at the UIC.

Forward and reverse reads were merged using the software package PEAR.^24^ Merged reads were trimmed to remove ambiguous nucleotides, primer sequences, and trimmed based on the quality threshold of *p* = 0.01. Merged reads that lacked either primer sequences or reads that were shorter than 225 bases were discarded. Chimeric sequences were identified and removed using the USEARCH algorithm with comparison to the SILVA v132 reference sequence database.^25^, ^26^ Amplicon sequence variants (ASVs) were identified using DADA2.^27^ The representative sequences for each ASVs were annotated using the Naïve Bayesian classifier included in DADA2 with the SILVA v132 reference sequence database.^28^ Basic annotation pipelines were performed by the Research Informatics Core at the UIC.

### 2.3 Outcomes

Primary outcome measures were associations between the microbiome (load, diversity, and pathogenic shift), HbA1c, and Vitamin C levels in DFU, IDS, and NDS samples. Secondary outcome measures examined the association between HbA1c, Vitamin C, wound size, and ulcer chronicity.

### 2.4 Statistical analysis

Statistical data analysis was performed with SPSS 25.0 (International Business Machines Corporation). Microbial communities were analyzed via 16S rRNA gene amplicon sequencing, and domain‐level qPCR of the 16S rRNA gene was utilized to determine bacterial load. Microbial community structure visualizations (multidimensional scaling) were performed in the software package Primer7,^29^ and statistical analyses were performed in the software package OriginPro 2015. Communities were analyzed for significant differences in α‐diversity and community structure between groups and depicted by multidimensional scaling plots and heat maps of the most common taxa. Analysis of similarity (ANOSIM) and Mann–Whitney nonparametric tests were performed to assess statistical significance (*p* < 0.05).

For patient characteristics, wound characteristics, and the dietary assessment, differences were assessed using ANOVA, Fisher's exact test, Mann–Whitney *U* test, *χ* ^2^ test, and multiple linear regression analysis.

## 3 RESULTS

The study included a total of 42 patients: 25 patients with DFU (7 patients with cHbA1c and 18 patients with uHbA1c), and 17 gender‐ and age‐matched controls (±4 years) without diabetes or ulcers. The characteristics of the study population are summarized in Table 1. The prevalence of Vitamin C deficiency in the DFU sample was 41.8% and 0% in the control group without diabetes or ulcer. While Vitamin C levels were comparable between those with well‐controlled diabetes and nondiabetic controls, significantly lower Vitamin C levels were observed in the uHbA1c group compared to nondiabetic controls (*p* = 0.002; Table 2). Larger wound sizes were noted in the uHbA1c group compared to the cHbA1c group (*p* = 0.46; Table 2). Mean ulcer duration at the time of enrollment was higher in the uHbA1c group (*p* = 0.28; Table 2). A simultaneous multiple regression was performed to evaluate how well wound surface area could be predicted from HbA1c levels, Vitamin C levels, age, body mass index, gender, and current tobacco use (Table 3). The overall regression was not statistically significant, *F*(6,29) = 0.984, *p* = 0.454.

**Table 1:** Patient characteristics at encounter

	Control (*n* = 17)	cHbA1c (*n* = 7)	uHbA1c (*n* = 18)	*p* Value
Age	55.3 ± 11.8	58.3 ± 9.2	53.0 ± 10.1	0.53
Body mass index	29.6 ± 4.9	31.3 ± 3.9	34.0 ± 9.1	0.21
Male (%)	15 (88.2)	6 (85.7)	14 (77.8)	0.70
Tobacco use (%)	1 (6.7)^a^	3 (42.9)	4 (22.2)^a^	0.16

*Note*: Data are reported as means ± standard deviation for continuous variables or as numbers (%) for categorical variables.

Abbreviations: cHbA1c, controlled hemoglobin A1c; uHbA1c, uncontrolled A1c.

^a^ Three patients in the control group and two patients in the uHbA1c group declined to provide their history of tobacco use.

**Table 2:** Blood test values and wound characteristics

	Control (*n* = 17)	cHbA1c (*n* = 7)	uHbA1c (*n* = 18)	Control_cHbA1c *p* Value	Control_uHbA1c *p* Value	cHbA1c_uHbA1c *p* Value
HbA1c (%)	5.4 ± 0.5	7.0 ± 0.6	10.2 ± 1.6	<0.001*	<0.001*	<0.001*
Vitamin C (mg/dl)	0.8 ± 0.4	0.7 ± 0.7	0.4 ± 0.3	0.62	0.002*	0.23
Wound size (cm^2^)	N/A	0.9 ± 0.8	3.0 ± 4.7	N/A	N/A	0.46
Mean ulcer duration at enrollment (weeks)	N/A	42.0 ± 41.9	52.3 ± 97.5	N/A	N/A	0.28

*Note*: Data are reported as means ± standard deviation.

Abbreviations: cHbA1c, controlled hemoglobin A1c; N/A, not applicable; uHbA1c, uncontrolled HbA1c.

* Statistically significant.

### 3.1 Quantitative analysis of microbial abundance

**Figure 1:**
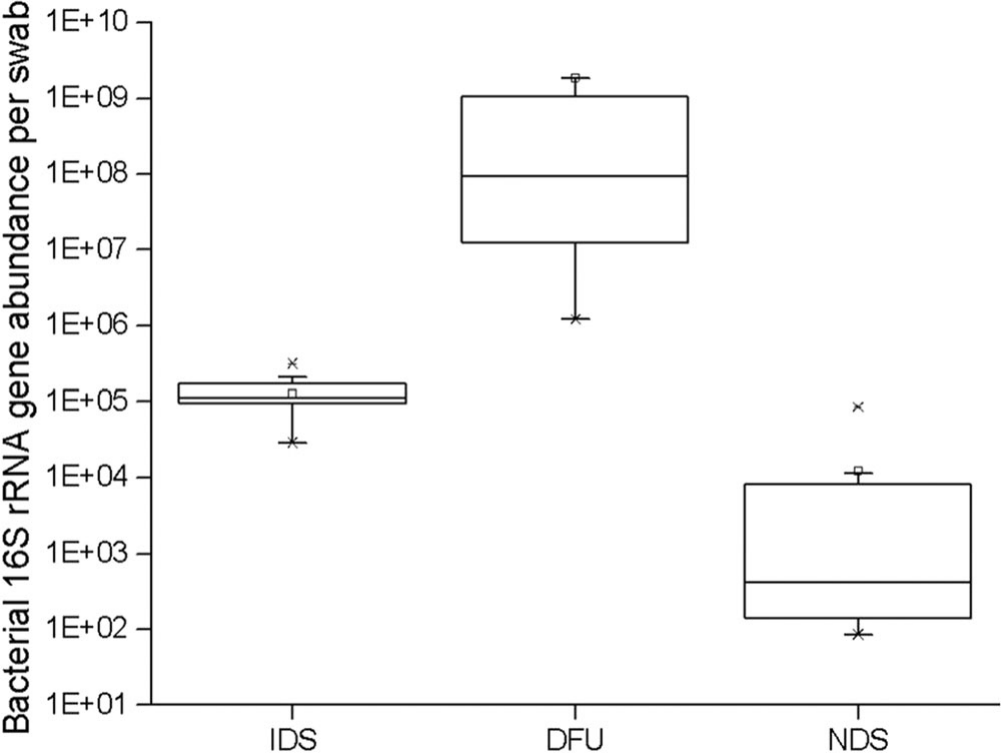
Bacterial load by ulcer status. Absolute bacterial abundance was measured using quantitative PCR targeting bacterial 16S rRNA genes. Box plots show the distribution of bacterial abundance by sampling location, with transverse lines representing the medians. The distributions are significantly different (Mann–Whitney test; *p* < 0.0004 for all pairwise comparisons; IDS, *n* = 11; DFU, *n* = 22; NDS, *n* = 9). DFU, diabetic foot ulcer; IDS, intact diabetic skin; NDS, nondiabetic; PCR, polymerase chain reaction; rRNA, ribosomal RNA.

Bacterial loads, as assessed by qPCR with domain‐level primers targeting 16S rRNA genes, were 4–5 orders of magnitude higher in DFU samples than in IDS or NDS samples. DFU samples (*n* = 22) had an average load of 1.89 × 10^9^ (±6.05 × 10^9^) copies/swab relative to averages of 1.31 × 10^5^ (±8.09 × 10^4^) copies/swab for IDS (*n* = 11) and 1.23 × 10^4^ (±2.78 × 10^4^) copies/swab for NDS (*n* = 9). Bacterial loads were significantly different between all groups, as assessed by a nonparametric Mann–Whitney *U* test (*p* < 0.0004 for all pairwise comparisons; Figure 1).

To determine if bacterial loads differed by HbA1c control, bacterial abundances in DFU from uHbA1c (*n* = 17) were compared to bacterial abundances in DFU from cHbA1c (*n* = 6). DFU samples from cHbA1c individuals had an average bacterial load of 8.22 × 10^8^ (±1.43 × 10^9^), and DFU samples from uHbA1c individuals had an average bacterial load of 2.30 × 10^9^ (±6.84 × 10^9^); these groups were not significantly different (Mann–Whitney *U* test; *p* = 0.83).

A simultaneous multiple regression was performed to evaluate how well bacterial load could be predicted from HbA1c levels, Vitamin C levels, age, gender, and current tobacco use (Table 4). The overall regression was statistically significant, *F*(5,32) = 3.752, *p* = 0.009, with *R* = 0.608 and adjusted *R* ^2^ = 0.271. That is, when HbA1c levels, Vitamin C levels, age, gender, and current tobacco are used as predictors, about 27% of the variance in bacterial load could be predicted. HbA1c was significantly predictive of the bacterial load controlling for Vitamin C levels, age, gender, and current tobacco use, *t*(32) = 4.08, *p* < 0.001. For every one unit increase in HbA1c, bacterial load increased by 0.656 log(average copies/swabbed area) when controlling for other predictors (95% confidence interval = 0.328, 0.984).

Bacterial abundances in DFU from IR Vitamin C (*n* = 12) were compared to bacterial abundances in DFU from OOR Vitamin C (*n* = 10). DFU samples from IR Vitamin C individuals had a mean bacterial load of 3.04 × 10^9^ (±8.13 × 10^9^), and DFU samples from OOR Vitamin C individuals had a mean bacterial load of 5.08 × 10^8^ (±7.54 × 10^8^); these groups were not significantly different (Mann–Whitney *U* test; *p* = 0.82).

### 3.2 Analysis of microbial community structure

Analysis of microbial community structure was performed using short‐read 16S rRNA gene amplicon sequencing on an Illumina MiniSeq platform. This primer set employed (515F/806R), which is derived from the Earth Microbiome Project, is highly robust, has been widely used across many sample types,^21^ including skin samples,^30^ and has broad coverage across Bacterial and Archaeal taxa, but has limited taxonomic resolution below the level of genus.^21^, ^30^ A total of 3,160,729 sequencing clusters were generated for the study, with a mean of 35,513 sequences/sample (standard deviation = 26,003). For calculation of α‐diversity indices, data were rarefied to a depth of 7000 sequences/sample. Due to the limited taxonomic resolution at the taxonomic level of species, α‐diversity analyses were performed at the taxonomic level of genus. Significantly fewer taxa were observed in DFU samples compared to IDS and NDS samples (Mann–Whitney *U* test, *p* < 0.01). There were also significantly fewer genera detected in IDS samples compared to NDS samples (Mann–Whitney *U* test, *p* < 0.01; Table 5). Shannon indices, which account for both richness and evenness of microbial community structure, were lower in DFU samples compared to IDS and NDS samples, but the significance was more marginal (*p* = 0.12, *p* < 0.05, respectively; Table 5). Shannon diversity was not significantly different between IDS and NDS samples (Table 5). A simultaneous multiple regression was performed to evaluate how well the Shannon index could be predicted from HbA1c levels, Vitamin C levels, age, gender, and current tobacco use (Table 6). The overall regression was not statistically significant, *F*(6,59) = 0.818, *p* = 0.541.

**Table 3:** Multiple regression analysis summary for predicting wound surface area

Variable	*B*	95% CI	β	*t*	*p* Value
(Constant)	−2.892	[−13.332, 7.549]		−0.566	0.575
HbA1c levels (%)	0.453	[−0.067, 0.974]	0.356	1.783	0.085
Vitamin C levels (mg/dl)	0.725	[−2.007, 3.457]	0.113	0.543	0.591
Age	0.012	[−0.094, 0.118]	0.041	0.231	0.819
BMI	−0.029	[−0.223, 0.165]	−0.056	−0.303	0.764
Gender	0.068	[−2.708, 2.844]	0.009	0.050	0.960
Current tobacco use	2.170	[−0.810, 5.151]	0.295	1.489	0.147

*Note*: *R* ^2^ adjusted = −0.003. CI = confidence interval for *B*.

Abbreviations: BMI, body mass index; HbA1c, hemoglobin A1c.

**Table 4:** Multiple regression analysis summary for predicting bacterial load

Variable	*B*	95% CI	β	*t*	*p* Value
(Constant)	−2.070	[−8.314, 4.174]		−0.675	0.504
HbA1c levels (%)	0.656	[0.328, 0.984]	0.690	4.077	<0.001
Vitamin C levels (mg/dl)	0.842	[−0.713, 2.398]	0.191	1.103	0.278
Age	0.039	[−0.034, 0.111]	0.172	1.082	0.287
Gender	−0.383	[−1.894, 1.128]	−0.075	−0.517	0.609
Current tobacco use	1.533	[−0.320, 3.386]	0.280	1.685	0.102

*Note*: *R* ^2^ adjusted = 0.271. CI = confidence interval for *B*.

Abbreviation: HbA1c, hemoglobin A1c.

**Table 5:** Genus‐level α‐diversity analysis

	Richness (number of genera detected)	Shannon index (*H*′; log *e*)
IDS (*n* = 23)	31.4 ± 13.5	1.7 ± 0.8
DFU (*n* = 25)	16.6 ± 5.2	1.3 ± 0.5
NDS (*n* = 28)	45.3 ± 15.8	1.7 ± 0.8
*p* Values		
IDS_NDS	0.002*	0.66
DFU_IDS	<0.001*	0.25
DFU_NDS	<0.001*	0.12

*Note*: Data are reported as means ± standard deviation.

Abbreviations: DFU, diabetic foot ulcer; IDS, intact diabetic skin; NDS, nondiabetic skin.

* Statistically significant.

**Table 6:** Multiple regression analysis summary for predicting Shannon index

Variable	*B*	95% CI	β	*t*	*p* Value
(Constant)	2.411	[0.687, 4.135]		2.799	0.007
HbA1c levels (%)	−0.045	[−0.139, 0.048]	−0.149	−0.965	0.339
Vitamin C levels (mg/dl)	−0.212	[−0.817, 0.394]	−0.119	−0.700	0.487
Age	−0.005	[−0.024, 0.014]	−0.067	−0.490	0.626
Gender	−0.185	[−0.709, 0.339]	−0.090	−0.707	0.483
Current tobacco use	−0.440	[−0.975, 0.095]	−0.244	−1.647	0.105

*Note*: *R* ^2^ adjusted = −0.014. CI = confidence interval for *B*.

Abbreviation: HbA1c, hemoglobin A1c.

**Figure 2:**
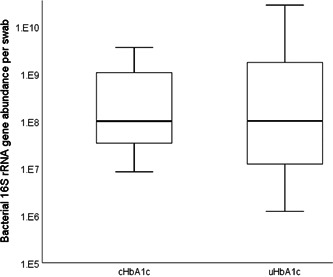
Bacterial load by glycemic control in DFU. Absolute bacterial abundance was measured using quantitative PCR targeting bacterial 16S rRNA genes. Box plots show the distribution of bacterial abundance by glycemic control in DFU, with transverse lines representing the medians. Means were not significantly different between cHbA1c and uHbA1c groups (Mann–Whitney *U* test, *p* = 0.83). cHbA1c, controlled hemoglobin A1c; DFU, diabetic foot ulcer; PCR, polymerase chain reaction; rRNA, ribosomal RNA; uHbA1c, uncontrolled HbA1c.​​​​​​

**Figure 3:**
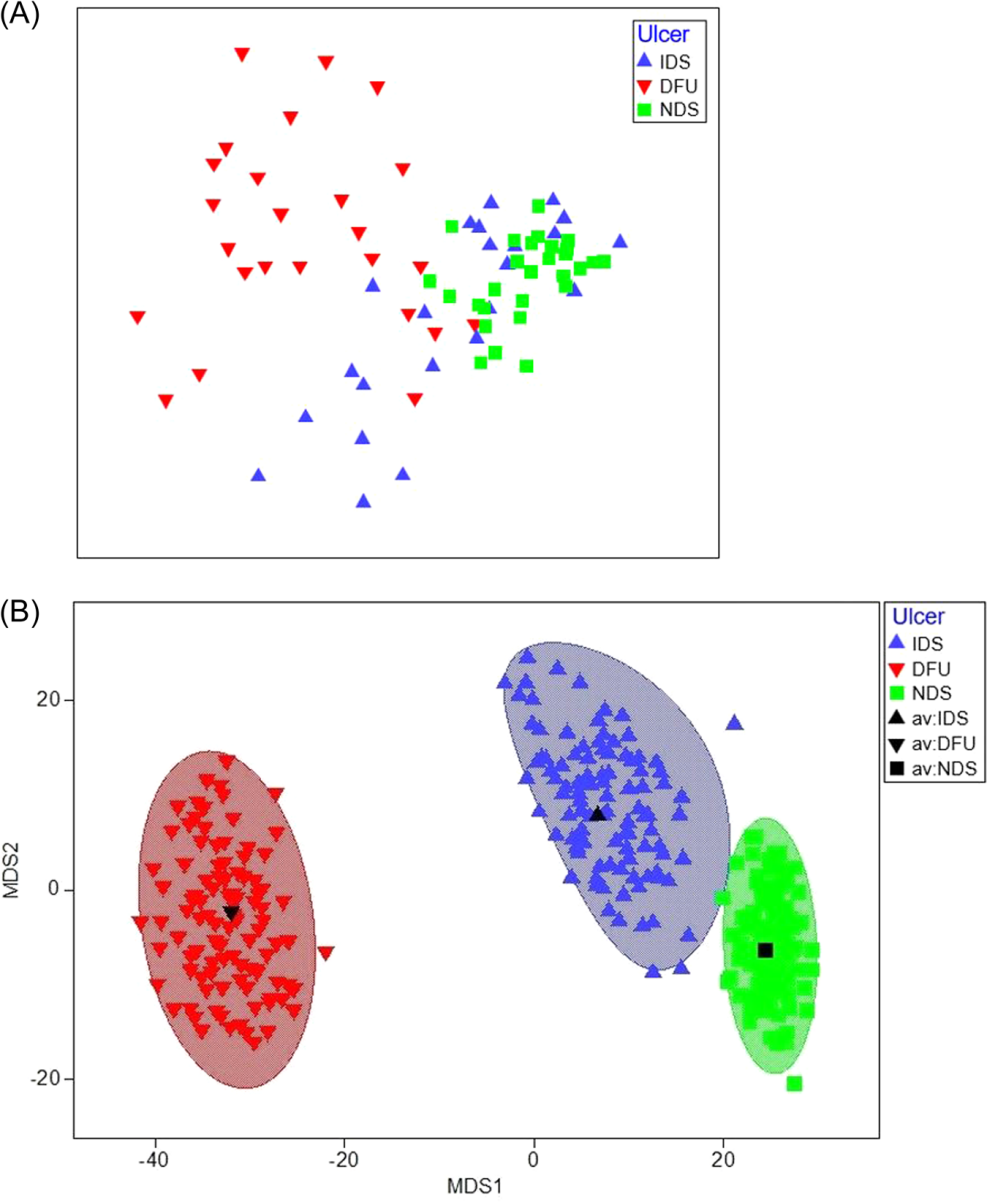
(A) Nonmetric MDS plot of ulcer groups. (B) Boot‐strapped averages of ulcer groups. Sequence data were visualized using metric MDS employing a distance matrix based on Bray–Curtis similarity at the taxonomic level of the genus. (A) Samples were color‐coded by group. Visually, DFU samples were separated from IDS and NDS samples, and this was confirmed by ANOSIM (DFU vs. IDS, *R* = 0.341/*p* = 0.001; DFU vs. NDS, *R* = 0.649/*p* = 0.001). IDS and NDS samples were also significantly different but at a smaller scale (ANOSIM IDS vs. NDS, R = 0.149/*p* = 0.001; 999 permutations). (B) mMDS of whole‐sample bootstrap averages with approximate 95% region estimates fitted to the bootstrap averages. The group means of DFU, IDS, and NDS are shown in black symbols and confirm the separation of DFU from IDS and NDS, and the greater similarity of IDS and NDS samples. ANOSIM, analysis of similarity; DFU, diabetic foot ulcer; IDS, intact diabetic skin; MDS, multidimensional scaling; NDS, nondiabetic; PCR, polymerase chain reaction.

**Figure 4:**
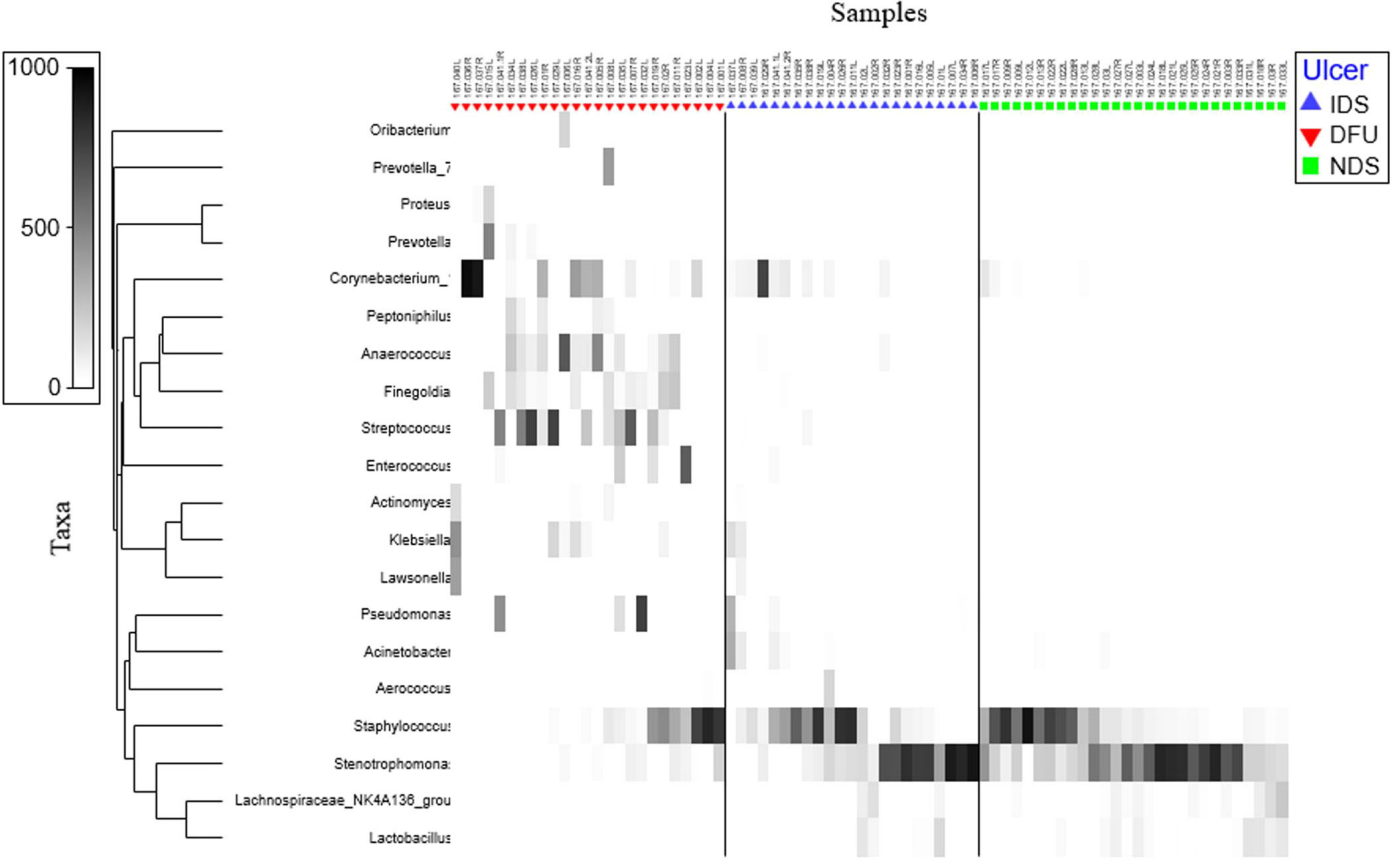
Heat map showing the relative abundance of the 20 most abundant taxa in IDS, DFU, and NDS samples. DFU, diabetic foot ulcer; IDS, intact diabetic skin; NDS, nondiabetic.

The overall microbial community structure in DFU samples was distinct from that observed in IDS and NDS samples (Figure 2A,B). Significant differences were observed between the three sample groups (one‐way ANOSIM global *R* = 0.383/*p* = 0.001; Figure 2A). However, no global significant differences were observed for HbA1c and Vitamin C levels using two‐way crossed ANOSIM across ulcer status (*R* < 0.013/*p* > 0.375). Although bacteria from the genera *Stenotrophomonas* and *Staphylococcus* were most abundant in IDS and NDS samples, a broader range of abundant taxa were observed in DFU samples, including putative anaerobes and pathogens, such as bacteria from the genera *Proteus*, *Anaerococcus*, *Finegoldia*, *Streptococcus*, and *Klebsiella* (Figure 3). No single putative pathogen was dominant in all DFU samples, but most DFU samples had lower relative abundances of *Stenotrophomonas* and *Staphylococcus* and higher relative abundance of putative pathogens relative to NDS and IDS samples (Figure 4).

### 3.3 Vitamin C dietary assessment survey

Forty survey results were included. Four of 17 (17%) patients with DFU reported dietary restrictions due to their diabetes. Nine of 17 (52.9%) patients with uHbA1c and two of seven (28.6%) with cHbA1c did not believe they met the daily recommended intake (DRI) of fruits. Seven of 17 (41.2%) patients with uHbA1c and 3 of 7 (42.9%) with cHbA1c did not believe they met the DRI of vegetables. Six of 11 (55%) patients with DFU who did not believe they met the DRI of fruits had Vitamin C levels that were OOR compared with 4 of 12 (33%) patients with DFU who believed they met the DRI. The correlation between monthly FV intake and Vitamin C levels for patients with DFU, cHbA1c, and uHbA1c were *R* ^2^ = 0.14 (*p* = 0.08), 0.44 (*p* = 0.15), and 0.01 (*p* = 0.69), respectively.

## 4 DISCUSSION

We observed characteristic features of DFU‐associated microbial communities, including reduced α‐diversity and dramatically and significantly increased bacterial load, relative to IDS and NDS samples. Similar results have been observed previously. Gontcharova et al.^31^ observed a significantly lower diversity of bacteria in DFU compared to IDS, but did not look at intact skin from nondiabetic patient controls. They also did not evaluate for associations with clinical factors. Prior studies have shown that the DFU microbial community is heterogeneous and dominated by bacteria from the genus *Staphylococcus*.^32^ In our study, the relative abundance of *Staphylococcus* and *Pseudomonas* was higher in IDS and NDS samples relative to DFU, a possible indication that these bacteria are not always contributors to or drivers of the pathogenesis of poorly healing DFUs. As these organisms are present in the skin microbiome of healthy individuals, their presence alone is not indicative of a health concern. However, it is also possible that these bacteria do not need to be highly abundant to inhibit DFU healing. Additional microbiological analyses are needed to assess the effect of bacterial loads on DFU healing. Xu et al.^33^ found that bacterial load can have a strong inverse relationship with DFU healing. Their results showed that for each log order of colony‐forming unit increase, ulcer healing was delayed by 44%. However, the investigators did not examine the relative abundance of each bacterium. Poorly healing DFU can be characterized by biofilms, or accumulations of microorganisms, which are diverse and complex.^34^ Quantitative load assessments of each bacterium would help characterize the effect of bacterial loads on DFU healing.

Notable putative pathogens that were more prevalent in DFU samples in this study, as compared to IDS and NDS samples, included a polymicrobial community of organisms, including those from the Gram‐positive genera *Actinomyces, Anaerococcus, Finegoldia, Streptococcus*, and *Enterococcus*, some of which have been shown to being pathogenic or opportunistic pathogens.^35^ *Actinomyces* are anaerobic, Gram‐positive bacteria that have been implicated in endodontic infections.^36^ *Anaerococcus* are another anaerobic, Gram‐positive bacteria commonly associated with gastrointestinal and urogenital disorders.^37^ *Finegoldia* are also anaerobic, Gram‐positive bacteria that induce inflammation by activating neutrophils.^38^ *Streptococcus* are aerobic, Gram‐positive bacteria that are commensal and pathogenic. They are associated with a number of disorders, including wound and skin infections, dental caries, and pneumonia.^39^ A recent study by Eydou et al.^40^ found that Vitamin C is associated with decreased *Streptococcus* growth and biofilm formation in specimens obtained from dental caries. Furthermore, it was found that the effects of Vitamin C were superior to gentamicin.^41^ Finally, *Enterococcus* are facultatively anaerobic, Gram‐positive bacteria that serve as a major causative agent of infections due to their multidrug resistance.^41^ Anaerobic bacteria are prevalent in DFU, but many may go undetected with standard culture swabs.^42^ We note, however, that not all members of these bacterial genera are pathogens, and that future studies employing long‐read amplicon sequencing and shotgun metagenome sequencing will be needed to fully evaluate the taxonomy and functional capabilities of these organisms in the wound environment.

Infected, nonhealing DFUs possess a mixed microbial community characterized by elevated microbial load, decreased microbial diversity, and community shift towards putatively pathogenic taxa.^43^ Historically, cultivation‐dependent analyses of DFU microbial community structure favored invasive tissue biopsies over swabs.^44^ However, cultivation approaches will also be limited by a priori knowledge of ideal growth conditions for the polymicrobial community and may miss substantial microbial diversity. Cultivation‐independent approaches are essential for capturing simultaneously aerobic/microaerophilic/anaerobic microorganisms present in the wound environment. Cultivation‐independent molecular tools have been recently used for the characterization of DFU‐associated microbial community structure. Travis et al.^35^ observed that wound swabs could effectively be used for DFU microbial community analyses when coupled with genomic DNA extraction followed by high‐throughput sequencing of microbial 16S rRNA gene amplicons. Such an approach has multiple advantages, due to less invasive sampling and a broader target range of known and potential pathogens by avoiding the need for cultivation. In addition, qPCR can be employed to quantify microbial load using assays targeting microbial rRNA genes. As a result, cultivation‐independent analyses based on 16S rRNA gene amplicon sequencing are replacing cultivation‐based swab analyses.^45^ Future approaches may also utilize shotgun metagenome sequencing to avoid targeted amplification of single genes, thereby providing more functional information including the presence or absence of pathogenicity genes and antibiotic resistance genes. Transcriptional profiling may also be employed to identify expressed genes of pathogens within the wound environment.

In this study, metric multidimensional scaling plots showed significantly different bacterial communities between the skin types, and the difference was greater with DFU. We further stratified DFU patients into controlled and uncontrolled HbA1c groups to determine if a relationship between glycemic control and bacterial characteristics exists. We observed that while DFU bacterial loads were lower in the cHbA1c group relative to those in the uHbA1c group, the difference was not significant. However, HbA1c was significantly predictive of bacterial load when controlling for Vitamin C levels, age, gender, and current tobacco use, where for every one unit increase in HbA1c, the bacterial load increased by 0.656 log(average copies/swabbed area). This is the first study to examine such a relationship.

Similar to other studies, we found Vitamin C deficiency to be more prevalent in the DFU population, 41.8% (10/24) compared to 0% (0/17) in the control group without diabetes or ulcer. These findings are consistent with the retrospective study performed by Brookes et al.^6^ involving 48 patients with DFUs, which found that 58.7% of patients had a Vitamin C deficiency, 30.4% of which had a severe deficiency and the risk of amputation was associated with Vitamin C (*p* < 0.01). Likewise, Pena et al.^5^ observed suboptimal levels of Vitamin C in 73% of patients with DFU. The role that Vitamin C may play in facilitating glycemic control warrants further investigation as Vitamin C deficiency was significantly more prevalent in the uHbA1c compared to cHbA1c group. Similarly, prior randomized controlled studies suggest that Vitamin C reduces glucose concentrations in patients with type 2 diabetes and on interventions lasting greater than 30 days.^12^

A recent pilot randomized, comparative study found supplementation with Vitamins C and E to be associated with increased healing of DFU at 12 weeks.^46^ However, the effect of Vitamin C supplementation on wound healing processes in the body is not known. In addition, Vitamin C kinetics in relation to the status of microbiomes has not been examined in the lower extremity. While microbial communities did not differ in the two Vitamin C groups, significantly lower Vitamin C levels were observed in patients with DFU and uncontrolled HbA1c levels. Future studies may examine factors that can predispose individuals with DFU to Vitamin C deficiency and the effect on healing rates.

The American Diabetes Association recommends eating nutrient‐dense foods and restricting carbohydrate intake to delay or prevent complications of diabetes.^47^ Results of the dietary assessment survey in this study found that only one‐sixth of patients with DFU reported following a diabetic diet. Furthermore, over 40% of patients with DFU reported not meeting the DRI of FV. While it was not statistically significant, a greater proportion of patients with DFU who believed they met the DRI of FV had cHbA1c, suggesting a possible protective role of FV intake on glycemic control. There was a positive correlation between reported monthly FV intake and Vitamin C levels in the cHbA1c group, suggesting that FV intake may increase Vitamin C levels.

A limitation of the study is the small sample size. Recruitment was suspended due to the COVID‐19 pandemic and the subsequent restriction of hours and access to the RFUHC in North Chicago and Libertyville. Patients could not be recruited for months after March 2020. By the time the restriction was lifted, S. J. G. was no longer employed at the UIC lab where the microbial analyses were performed. With only seven patients in the cHbA1c group, comparisons of bacterial abundance with cHbA1c versus uHbA1c are affected by high within‐group variability, and such analyses may mask true differences. However, the data varied enough to show significance, despite the small sample size, and warrants further studies involving larger sample sizes.

Another potential limitation is inconsistency associated with the collection of serum Vitamin C levels. The patient preparation protocol from Quest Diagnostics for Vitamin C lab testing preferred overnight fasting and avoidance of fruit or Vitamin C supplementation within 24 h of blood collection. However, there were no supportive data provided by Quest Diagnostics or in the literature for the above‐mentioned preference. Prior studies assessing Vitamin C levels and glucose risk/diabetes management have not required overnight fasting or avoidance of fruit; therefore, Quest Diagnostic's preference was not used as exclusion criteria for blood collection.^5^, ^48^ Moreover, subjects were recruited in a random, consecutive manner. Potential subjects, not having previous knowledge about the study, will likely consume foods based on their dietary habits. In other words, people who generally consume lots of fruits and vegetables would likely have consumed fruits/vegetables 24 h prior and the ones who generally do not, likely would not have. Survey results support this; of the five patients with DFU who reported not eating fruit within 24 h of the visit, four reported not meeting the DRI of FV and three had Vitamin C levels that were OOR. The one patient who was not OOR reported taking Vitamin C supplements. Capturing the subjects in their “natural state” may allow for a more accurate assessment of the patient's Vitamin C levels.

Finally, previous studies have found differences in skin microbiota in males compared to females.^49^ For instance, Jnana et al.^49^ found an increase in facultative anaerobes such as *Proteus* and *Burkholderia* in males compared to females. Gender differences could not sufficiently be accounted for in this study due to the small female cohort (7/42). Therefore, gender differences may be a potential confounding factor in this study. Furthermore, while there is a potential technical limitation due to swabbing, it is less invasive than collecting a tissue specimen and therefore commonly used in microbial analysis studies.^15^ In addition, while the size of the ulcers swabbed was variable and can affect bacterial load analyses, the Levine technique was performed to standardize the swabs. Lastly, while there are technical limitations with the use of qPCR for bacterial load analysis, using qPCR with high‐throughput sequencing increases accuracy.^50^

In summary, the results of this study demonstrate an altered microbial community structure in DFU relative to IDS and NDS, with a dramatically elevated bacterial load. Vitamin C levels were also noted to be significantly lower in the uHbA1c group versus nondiabetic controls (*p* = 0.002). To our knowledge, this study is the first to assess the association between glycemic control, Vitamin C, and microbial community structure. While we did not find evidence for a strong relationship between Vitamin C or HbA1c and microbial community structure, the microbial community structure differs in DFU, IDS, and NDS. The association between diabetes, DFUs, and Vitamin C deficiency warrants further investigation with larger sample sizes.

## AUTHOR CONTRIBUTIONS

**Khanh Phuong S. Tong**: Conceptualization; data curation; formal analysis; funding acquisition; investigation; methodology; resources; validation; writing—original draft; writing—review and editing. **Stefan Green**: Formal analysis, methodology, writing—review and editing. **Jacquelyn Ortiz**: Investigation; methodology; resources; writing—review and editing. **Stephanie Wu**: Conceptualization; funding acquisition; methodology; supervision; writing—review and editing. All authors read and approved the final manuscript. Khanh Phuong C. Tong had full access to all of the data in this study and takes complete responsibility for the integrity of the data and the accuracy of the data analysis.

## CONFLICT OF INTEREST

The authors declare no conflict of interest.

## TRANSPARENCY STATEMENT

Khanh Phuong S. Tong affirms that this manuscript is an honest, accurate, and transparent account of the study being reported; that no important aspects of the study have been omitted; and that any discrepancies from the study as planned (and, if relevant, registered) have been explained.

## Data Availability

The data that support the findings of this study are available from the corresponding author upon reasonable request.

## APPENDIX A

**DIETARY ASSESSMENT SURVEY**

1. Do you have any dietary restrictions (including food allergies)?
2. When was the last time you ate or drank anything, other than water?
3. Do you believe you meet the daily recommended intake of **fruits** (1.5–2.0 cups)?
  Yes or No
  If you circled “No,” why not? **Circle one or more**.
  Fruit has too much sugar
  Cost
  Taste
  Other: ______________________________
4. Do you believe you meet the daily recommended intake of **vegetables** (2–3 cups)?
  Yes or No
  If you circled “No,” why not? **Circle one or more**.
  Preparation time
  Cost
  Taste
  Other: ______________________________
5. During the past month, how often did you eat **fruit**? Include fresh, frozen or canned fruit. Do not include juices. **Circle one**.
  Never
  1 time last month
  2–3 times last month
  1 time per week
  2 times per week
  3–4 times per week
  5–6 times per week
  1 time per day
  2 or more times per day
6. Have you eaten fruit(s) within the last 24 hours? If yes, what fruit(s)?
7. During the past month, how often did you eat peach, kiwifruit, orange, apple, lemon, strawberry, papaya, and/or grapefruit? Circle one.
  Never
  1 time last month
  2–3 times last month
  1 time per week
  2 times per week
  3–4 times per week
  5–6 times per week
  1 time per day
  2 or more times per day
8. During the past month, how often did you eat **vegetables**? Include fresh, frozen and canned vegetables. Circle one.
  Never
  1 time last month
  2–3 times last month
  1 time per week
  2 times per week
  3–4 times per week
  5–6 times per week
  1 time per day
  2 or more times per day
9. During the past month, how often did you eat red or yellow sweet peppers, mustard spinach, broccoli, kale, peas, and/or brussel sprouts? Include fresh, frozen and canned. Circle one.
  Never
  1 time last month
  2–3 times last month
  1 time per week
  2 times per week
  3–4 times per week
  5–6 times per week
  1 time per day
  2 or more times per day
10. During the past month, how often did you drink 100% pure fruit juices such as orange, grapefruit, mango, apple, grape, pineapple, and tomato juices? Do not include fruit‐flavored drinks with added sugar or fruit juice you made at home and added sugar to. Circle one.
  Never
  1 time last month
  2–3 times last month
  1 time per week
  2 times per week
  3–4 times per week
  5–6 times per week
  1 time per day
  2–3 times per day
  4–5 times per day
  6 or more times per day
11. Do you take **vitamins or supplements**? Yes or No.
  If yes, which vitamins/supplement(s)?
  How often? Circle one.
  1–3 tablets per week
  4–6 tablets per week
  1 tablet per day
  2 tablets per day
  3 or more tablets per day
12. Did you take a vitamin/supplement within the last 24 hours? If yes, which vitamins/supplements?
